# Has the airway microbiome been overlooked in respiratory disease?

**DOI:** 10.1186/s13073-015-0184-9

**Published:** 2015-06-28

**Authors:** Olawale Salami, Benjamin J Marsland

**Affiliations:** Department of Biology and Medicine, Service de Pneumologie, CHUV-UNIL, Lausanne, Switzerland

## Abstract

The respiratory disease field is changing because of recent advances in our understanding of the airway microbiome. Central to this is dysbiosis, an imbalance of microbial communities that can lead to and flag inflammation in the airways. The increasing momentum of research in this area holds promise for novel treatment strategies.

## The airway microbiome is changing the respiratory disease field

In the past decade, a multiplicity of evidence has emerged in support of the central role of the human microbiome in the etio-pathogenesis of a wide variety of diseases. Work in this area has been aided by the increasing application of culture-independent microbial detection tools, such as high-throughput sequencing and metagenomics. Owing to the relative accessibility of the upper airway, previous attention has focused on sampling microorganisms from this region as representative of the airway microbiota. Recently, however, characterization of the lower-airway microbiota within the context of respiratory health and disease has prompted a shift away from the age-long credo of sterility of the lower airways, towards an overall consideration of the vital role of host-microbiota interactions in respiratory health.

Concurrently, interest on the role of non-bacterial members of the microbiota — viruses and fungi — is beginning to emerge. Notably, a complex mixture of bacterial and fungal communities has been identified in the airways of patients with cystic fibrosis. In addition, human tissue surfaces, including the lungs, harbor diverse viral populations [1]. Numerous gaps remain in our understanding of how ecological balance is achieved and maintained within co-existing viral, fungal and bacterial communities. As is now well established within the context of intestinal diseases, the local microbiota is a dominant player in maintaining a healthy homeostasis or in driving the development of disease. In the respiratory field, this emerging paradigm could have a significant impact on the goal of moving from symptomatic treatment to cures.

## Topographical and ecological perspective of the airway microbiome

The human respiratory tract is not homogenous but is comprised of microhabitats that are distinguished by different constitutive physico-chemical properties, such as pH, temperature, oxygen concentration, mucus production and nutrient availability, all of which could potentially shape the composition of the airway microbiota [2]. The presence of different microbial species in the airways may also be an indication of physiochemical property gradients within the airway microhabitats, allowing different regions of the airways to meet the survival needs of different microbial species. Other factors, such as inspiratory influx of nasopharyngeal and/or oral microbiota, or efficiency of physical elimination by coughing and micro-aspiration of gastro-esophageal contents, may also contribute to the topographical heterogeneity of the airway microbiota.

The currently proposed concept of a ‘healthy’ airway microbiota in humans, consisting of several putative airway pathogens, contradicts established models of infection in sterile tissues. For instance, the six dominant genera in the upper airway microbiota in children have been shown to be *Haemophilus*, *Streptococcus*, *Corynebacterium*, *Staphylococcus*, *Moraxella*, and *Alloiococcus* [3], members of which are commonly implicated in respiratory tract infections. Similarly, studies in the bronchoalveolar lavage fluid of healthy adults have shown a high frequency of potential ‘pathogens’, including *Pseudomonas*, *Streptococcus*, *Prevotella*, *Fusobacterium*, *Haemophilus*, *Veillonella*, and *Porphyromonas* [4]. In addition, asymptomatic nasopharyngeal colonization with *Streptococcus pneumonia*e, which has been shown to predate invasive pneumococcal disease in children, is characterized by a dysbiotic precursory outgrowth of *S. pneumoniae* within the nasopharyngeal microbiota [5].

Highlighting the role of the microbiota in shaping airway immunity, Teo and colleagues [3] recently examined the microbiota from nasopharyngeal aspirates collected within the first year of life from a cohort of 234 children, using 16S rRNA gene sequencing. They observed that dynamic transitions occurred within the nasopharyngeal microbiota, with early colonizers, *Staphylococcus* and *Corynebacterium*, replaced at one year by *Alloicoccus* and *Moraxella*. Importantly, these authors found significant associations between nasopharyngeal colonization by *Haemophilus*, *Streptococcus* and *Moraxella* and increased risks of virus-associated acute respiratory illnesses [3]. The subsequent risk of early childhood asthma was elevated as a consequence of microbiota-associated susceptibility to acute viral illnesses, especially asymptomatic streptococcal colonization in the first year of life [3]. There are two key lessons that can be drawn from this study. First, the composition of microbial communities within the nasopharynx in early life can determine susceptibility to viral infections of the lower airways. Second, and perhaps more importantly, early life changes in host–microbiota interactions may lead to long-term predisposition to airway inflammation. Therefore, within the context of airway infection, dynamic inter-species interactions that exist within poly-microbial niches in the airway microbiota may dictate the pathogenic potential of any single bacterial species, tipping the balance towards infection and chronic airway inflammation.

## Microbial dysbiosis in airway disease

Previous studies have suggested a link between microbial dysbiosis, defined as a perturbation in the structure of complex microbial communities [6], and the pathogenesis of chronic lung diseases, including chronic obstructive pulmonary disease [4] and allergic asthma [7]. Within the context of asthma, it is currently unclear whether dysbiosis represents an underlying susceptibility to airway colonization by certain bacteria or if the microbiota directly drives the aberrant inflammatory immune responses that characterize asthma. From an ecological standpoint, however, asthma-associated dysbiosis could result from a breakdown in balanced inter-species interactions within the lung microbiota that selects for outgrowths of dominant members, which could explain the association between antibiotic use in early life and subsequent risk of asthma [8]. A similar scenario of dysregulated ecological balance within microbial niches may underlie aberrant inflammation and chronicity associated with other chronic lung disorders, such as cystic fibrosis and idiopathic pulmonary fibrosis.

Low diversity of the gut microbiota in early infancy has been shown to be an important risk factor for the development of allergy. Furthermore, epidemiological evidence suggests an association between growing up in a farm environment, with early life exposure to barn-derived microbes and unpasteurized milk consumption, and decreased risk of developing allergy later in life [9]. Evidence from experiments in mouse models also alludes to the relevance of early life exposure in the education of developing host immune cells to allow tolerance of aero-allergens [10]. The protective effect of early life microbial exposure in the setting of allergic airway inflammation fits with the current paradigm of critical developmental windows in early life, when dysregulated host–microbiota interactions result in life-long sequelae.

## Current challenges and future directions

A substantial body of evidence now supports the concept that dysregulation of host–microbiota crosstalk at body surfaces may underlie chronic inflammatory disorders. From a clinical perspective, there is growing interest in the prospects of translating the results of airway microbiome analyses into practical patient-management tools for lung diseases; but in order to accelerate translational prospects of airway microbiome research, several challenges will need to be addressed. First, a harmonization of methodologies for airway sample collection, processing and analysis is required to provide comparable datasets. In addition, more work is needed to elucidate the broader interactions between bacterial, viral and fungal components of the microbiota and how they impact airway disease pathogenesis. Finally, a functional characterization of the airway microbiota using animal models and proteomic, transcriptomic and metabolomic approaches should provide crucial insights into how differential characteristics of the airway microbiota impact respiratory health. Specifically, these tools may provide insights into the predominant transcriptional and metabolic pathways, small molecules and metabolites within the microbiota that mediate host–microbiota and microbe–microbe interactions. Microbiota-derived metabolic pathways and metabolites could serve as future therapeutic targets in airway diseases.

A microbiota profile may serve as a future diagnostic or prognostic marker in airway inflammation. Similarly, changes in the microbiota could become indicators for monitoring airway disease progression and could guide therapeutic interventions. Approaches towards deliberate manipulation of the airway microbiota opens exciting possibilities for non-antibiotic-based treatment modalities in upper and lower respiratory tract infections. In addition, engineering the microbiota, by use of metabolites or probiotics during critical developmental windows in early life, may serve as an important prophylactic intervention to prevent chronic airway inflammation.
